# Immunoglobulin G4-related disease diagnosed by prostate biopsy: a case report

**DOI:** 10.1186/s13256-022-03611-4

**Published:** 2022-09-30

**Authors:** Shigeyuki Aoki, Shingo Morinaga, Naoki Kawai, Haruna Tanaka, Keiko Kanematsu, Nanami Tsuchiya, Sayuri Nonomura, Akiko Ozawa, Rie Imai, Ryoko Takahashi, Tomoko Sawada, Ruriko Futamachi, Yoshiaki Yamada

**Affiliations:** 1 The Department of Urology, Japan Community Health Care Organization Kani Tono Hospital, Gifu, Japan; 2 The Department of Clinical Laboratory, Japan Community Health Care Organization Kani Tono Hospital, Gifu, Japan; 3 The Division of Nursing, Japan Community Health Care Organization Kani Tono Hospital, Gifu, Japan; 4 The Division of Hospital and Clinic Coordination, Japan Community Health Care Organization Kani Tono Hospital, Gifu, Japan

**Keywords:** IgG4-related disease, IgG4-related prostatitis, Periaortitis, Prostate biopsy

## Abstract

**Background:**

Immunoglobulin G4-related disease is characterized by swelling of various organs throughout the body and nodules/hypertrophic lesions. However, its cause remains unknown. We report a case of immunoglobulin G4-related disease that was diagnosed based on the histopathological findings of prostate biopsy.

**Case presentation:**

A 72-year-old Japanese man had been treated by a nearby doctor for hypertension, but subsequently developed lower urinary tract symptoms and was prescribed an α1 blocker for 1 year. However, the patient was subsequently referred to our department because his symptoms did not improve. Prostate-specific antigen was 1.258 ng/ml; however, the nodule was palpable in the right lobe on digital rectal examination, and magnetic resonance imaging suggested Prostate Imaging and Reporting and Data System category 3. Therefore, transrectal prostate needle biopsy (12 locations) under ultrasound was performed. Histopathological examination revealed no malignant findings, although infiltration of lymphocytes and plasma cells, and partial fibrosis were observed. No remarkable findings of obstructive phlebitis were observed. Immunoglobulin G4-related disease was suspected, and immunoglobulin and immunoglobulin G4 immunostaining was performed. Immunoglobulin G4 positive plasma cells were observed in a wide range, immunoglobulin G4 positive cells were noted at > 10 per high-power field, and the immunoglobulin G4 positive/immunoglobulin G positive cell ratio was > 40%. Serum immunoglobulin G4 levels were high at 1600 mg/dl. Enhanced abdominal computed tomography findings suggested periaortitis. Additionally, multiple lymphadenopathies were observed around the abdominal aorta. The patient was accordingly diagnosed with immunoglobulin G4-related disease definite, diagnosis group (definite). We proposed steroid treatment for periaortic soft tissue lesions and lower urinary tract symptoms; however, the patient was refused treatment. A computed tomography scan 6 months after diagnosis revealed no changes in the soft tissue lesions around the aorta. Follow-up computed tomography examinations will be performed every 6 months.

**Conclusion:**

If immunoglobulin G4-related disease is suspected and a highly invasive examination is required for histopathological diagnosis, this can be performed by a relatively minimally invasive prostate biopsy for patients with lower urinary tract symptoms. Further evidence is needed to choose an optimal candidate for prostate biopsy for lower urinary tract symptoms patients with suspicion of immunoglobulin G4-related disease. For patients with lower urinary tract symptoms with immunoglobulin G4-related disease or a history, performing a prostate biopsy may avoid unnecessary treatment. However, if steroid therapy is ineffective, surgical treatment should be considered.

## Background

Immunoglobulin G4-related disease (IgG4-RD) was first reported in 2001 in patients with sclerosing pancreatitis and elevated serum IgG4 levels [1] . Subsequently, IgG4-RD was established as an unexplained disease with various systemic lesions [2] . Here, we report a case of IgG4-RD that was histopathologically diagnosed by prostate biopsy.

## Case presentation

A 72-year-old Japanese man had been treated by a nearby doctor for hypertension, but subsequently developed lower urinary tract symptoms (LUTS; lower abdominal discomfort and difficulty urinating) and was prescribed an α1 blocker for 1 year. However, the patient was subsequently referred to our department because his symptoms did not improve. His medical history included sinusitis and laparoscopic cholecystectomy. At the first visit, the patient’s height and weight were 162 cm and 66 kg, respectively. HIs blood pressure was 132/74 mmHg, pulse was regular at 63 beats/minute, and body temperature was 36.4 °C. The patient had no history of smoking or drinking. No specific abnormalities or neurological findings were noted at the initial physical examination. However, a digital rectal examination revealed a walnut-sized prostate without tenderness, with a palpable nodule on the right lobe.

Blood biochemistry and urinalysis revealed no abnormal findings, and the prostate-specific antigen (PSA) level was 1.258 ng/ml.

Abdominal ultrasonography revealed a prostate mass of 36 cc. The International Prostate Symptom Score (IPSS) was 20 points, IPSS-Quality of Life was 4 points, and uroflowmetry revealed a urine volume of 211 ml, maximum urine flow rate of 10.7 ml/s, and residual urine volume of 10 ml.

**Fig. 1:**
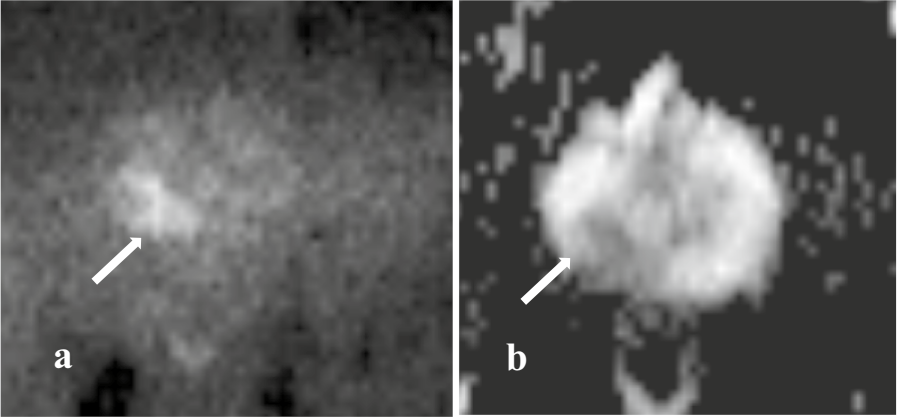
Pelvic MRI revealed a high-intensity area in the right lobe on DWI (**a**: white arrow) and a low-intensity area for ADC (**b**: white arrow)

Pelvic magnetic resonance imaging (MRI) revealed a high-intensity area in the right lobe on diffusion-weighted imaging (DWI) (Fig. 1a), and the apparent diffusion coefficient (ADC) in that area revealed a low signal (Fig 1b). No other abnormalities were detected in the search range. The radiologist’s interpretation result was according to Prostate Imaging and Reporting and Data System (PI-RADS) category 3.

Digital rectal examination and MRI findings suggested cT2a prostate cancer; therefore, a transrectal ultrasound-guided systematic biopsy (12 cores) was performed.

**Fig. 2:**
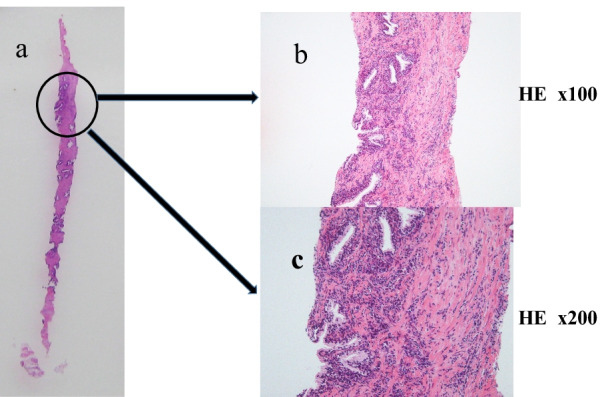
Pathological findings of the prostate biopsy specimen. The biopsy specimen revealed prostatitis and fibrosis. Lymphocytes/plasma cell-based chronic inflammatory cell infiltration is recognized. Focal inflammatory infiltrate with plasma cells. **a** Biopsy specimen. **b** HE staining. (× 100). **c** HE staining. (× 200)

**Fig. 3:**
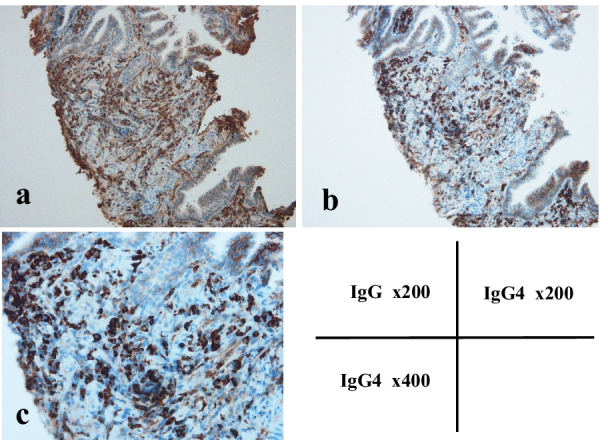
The immunohistochemical evaluation of the biopsy specimens indicated positive IgG and IgG4 immunostaining: IgG4+/IgG+ cell ratio was > 40%, and the number of IgG4+ plasma cells was > 10 per high-power field. **a** IgG immunostaining. (× 200). **b** IgG4 immunostaining. (× 200). **c** IgG4 immunostaining. (× 400)

A pathological examination revealed no malignancy and poor glandular formation, while only a dense lymphoplasmacytic infiltration but no obliterative phlebitis or storiform fibrosis was observed (Fig. 2a–c). The pathologist’s diagnosis was suspected IgG4-RD. Thus, we performed IgG and IgG4 immunostaining with suspicion of IgG4-RD. IgG4-positive (IgG4+) plasma cells were observed in a wide range, IgG4+ cells were noted at > 10 per high-power field, and the IgG4+/IgG-positive (IgG+) cell ratio was > 40% (Fig. 3a–c).

**Fig. 4:**
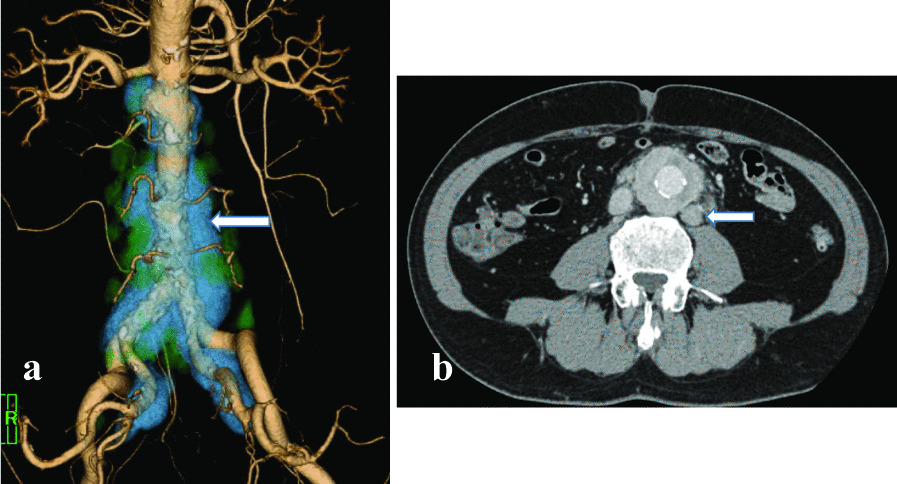
Abdominal enhanced CT revealed a soft tissue mass around the bilateral common iliac arteries from the abdominal aorta (**a** white arrow, 3D-CT). Additionally, multiple lymphadenopathy is observed around the abdominal aorta (**b** white arrow)

Serum IgG4 measurements and lung and abdominal computed tomography (CT) were performed for a definitive diagnosis of IgG4-RD. Serum IgG4 levels were high at 1600 mg/dl (reference range: 11–121–mg/dl). Pulmonary CT indicated no abnormal findings, and enhanced abdominal CT revealed a soft tissue mass around the bilateral common iliac arteries from the abdominal aorta, suggesting periaortitis (Fig. 4a). Additionally, multiple lymphadenopathies were observed around the abdominal aorta (Fig. 4b), and malignant lymphoma was suspected. Therefore, the soluble interleukin 2 receptor was measured and was within the normal range of 392 U/ml (reference range: 121–613 U/ml).

Based on the above findings, we diagnosed the patient with IgG4-RD definite, diagnosis group (definite).

Regarding treatment strategy, we decided that no invasive treatment was needed as no symptoms were associated with the periaortic lesions. We proposed steroid treatment for LUTS; however, the patient refused treatment because of concerns regarding the side effects associated with steroid treatment. An α1 blocker that had no effect on the patient was also discontinued, and a CT scan 6 months after diagnosis revealed no changes in the soft tissue lesions around the aorta. Follow-up CT examinations will be performed every 6 months.

## Discussion

The present case underscores the utility of an alternative prostate biopsy for a definitive diagnosis in patients with suspected IgG4-RD LUTS who have suffered from uncontrollable LUTS. IgG4-RD was first reported by Hamano *et al.* [1] in 2001, and was reportedly associated with autoimmune pancreatitis and IgG4. Histopathological examination revealed lymphocyte infiltration, IgG4+ plasma cell infiltration, and fibrosis, and was therefore defined as an unexplained disease with swelling, nodules, and hypertrophic lesions in various organs throughout the body [2] . The incidence is 0.28–1.08 per 100,000 [3] , and the disease reportedly occurs more often in men aged > 50 years [4] .

The diagnostic criteria for IgG4-RD were presented in 2011 and revised in 2020. That is, (Item 1) Clinical and radiological features: One or more organs show diffuse or localized swelling or a mass or nodule characteristic of IgG4-RD. In single organ involvement, lymph nodes swelling is omitted. (Item 2) Serological diagnosis: Serum IgG4 levels greater than 135 mg/dl. (Item 3) Pathological diagnosis: Positive for two of the following three criteria; (1) Dense lymphocyte and plasma cell infiltration with fibrosis; (2) Ratio of IgG4+ plasma cells/IgG+ cells greater than 40% and number of IgG4+ plasma cells greater than 10 per high powered field; (3) Typical tissue fibrosis, particularly storiform fibrosis, or obliterative phlebitis. A definite diagnosis group was defined as a case in which all three items were satisfied [5] . In our case, diffusely thickened soft tissue lesions were identified around the bilateral common iliac arteries from the abdominal aorta, thus satisfying Item 1. In addition, the high serum IgG4 level satisfied Item 2, and the histopathological findings indicated that conditions (1) and (2) of Item 3 were satisfied, so the patient was diagnosed as belonging to the definite diagnosis group.

Regarding diagnostic imaging in IgG4-RD, characteristic imaging findings have been reported for autoimmune pancreatitis and nephritis. However, no characteristic images have been reported to date for other organs, including the prostate [6]. In our patient, we decided to perform a prostate biopsy based on the palpable nodule on a digital rectal examination and PI-RADS category 3 lesion on MRI, despite serum PSA within the normal range. To the best of our knowledge, there are no studies reporting the correlation between serum PSA or digital rectal examination abnormalities and IgG4-RD.

Buijis *et al.* [7] reported that all nine patients of 117 IgG4-RD patients with pancreatic cholangitis who complained of LUTS had IgG4+ plasma cell infiltration in the prostate tissue on prostate biopsy. In addition, Uehara *et al.* [8] reported that prostate biopsy was performed in all patients with autoimmune pancreatitis with or without LUTS, and histological findings of IgG4-RD were observed in all six patients with LUTS. These reports have suggested that prostate biopsy may be useful for histopathological diagnosis of IgG4-RD with LUTS.

Differentiation of IgG4-RD from other similar diseases, such as sarcoidosis, multicentric Castleman disease, malignant lymphoma, granulomatosis with polyangiitis, and cancer, is necessary [9], so direct histological evidence must be obtained, and a biopsy of the primary affected lesion should be considered whenever possible for making a definitive diagnosis of IgG4-RD. However, histopathological examination of some lesions is not always feasible, and a highly invasive examination may be required depending on the target organ. In a retrospective study, Mizushima *et al.* [10] assessed the clinical course of patients with aortitis/periaortitis and periarteritis treated with IgG4-RD, only one of 40 patients received open retroperitoneal biopsy for histopathological diagnosis. Therefore, if IgG4-RD can be diagnosed using a relatively minimally invasive prostate biopsy, it would be of great benefit to the patient.

By investigating the possibility of prostate disease in male patients with suspected IgG4-RD, unnecessary invasive testing may be avoided. However, performing prostate biopsy for the histopathological diagnosis of patients without prostate disease is ethically problematic. Further evidence is needed to choose an optimal candidate for prostate biopsy for LUTS patients with suspicion of IgG4-RD.

To the best of our knowledge, only one case diagnosed with IgG4-RD based on the histopathological findings of prostate biopsy, which was performed with suspicion of prostate cancer, as in our case, has been recorded in Japan [11] .

Although there are many reports of IgG4-RD in Japan, studies are limited, and many aspects of the disease remain unknown. Moreover, not all cases are complicated by autoimmune pancreatitis, so diagnosis can be difficult [12] .

However, since there are many successful cases of steroid treatment, it is highly possible that invasive tests and unnecessary treatment/surgery can be avoided by understanding the concept of the disease. In patients with IgG4-RD or a history of LUTS, prostate biopsy should be actively considered to avoid unnecessary treatment/surgery.

## Conclusion

We reported a case of IgG4-RD based on histopathological findings of a prostate biopsy, which was performed with suspicion of prostate cancer.

If IgG4-RD is suspected and highly invasive examination is required for histopathological diagnosis, this can be performed by a relatively minimally invasive prostate biopsy for patients with LUTS. Further evidence is needed to choose an optimal candidate for prostate biopsy in LUTS patients with suspicion of IgG4-RD.

For LUTS patients with IgG4-RD or a history, performing a prostate biopsy may avoid unnecessary treatment. However, if steroid therapy is ineffective, surgical treatment should be considered.

## Data Availability

Not applicable.

## Abbreviations

IgG4-RD: Immunoglobulin G4-related disease
LUTS: Lower urinary tract symptoms
PSA: Prostate-specific antigen
MRI: Magnetic resonance imaging
PI-RADS: Prostate Imaging and Reporting and Data System
IgG4+: Immunoglobulin G4 positive
IgG+: Immunoglobulin G positive
CT: Computed tomography
IPSS: International Prostate Symptom Score
DWI: Diffusion-weighted imaging
ADC: Apparent diffusion coefficient
